# On the formation of seven-membered rings by arene-ynamide cyclization

**DOI:** 10.1007/s00706-018-2320-x

**Published:** 2018-11-16

**Authors:** Bogdan R. Brutiu, Wilhelm Andrei Bubeneck, Olivera Cvetkovic, Jing Li, Nuno Maulide

**Affiliations:** Institute of Organic Chemistry, University of Vienna, Währinger Strasse 38, 1090 Vienna, Austria

**Keywords:** Heterocycles, Strained molecules, Brønsted acid, Catalysis

## Abstract

**Abstract:**

A Brønsted acid-catalyzed selective arene-ynamide cyclization is described. This reaction proceeds via a keteniminium intermediate and enables the preparation of seven-membered ring enamide products. Mechanistic studies uncover an unusual product inhibition behavior.

**Graphical abstract:**

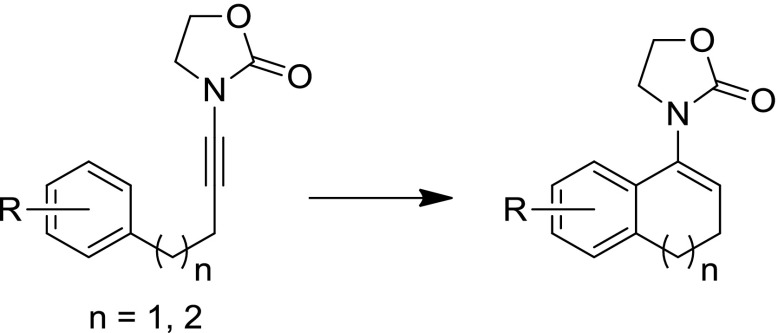

**Electronic supplementary material:**

The online version of this article (10.1007/s00706-018-2320-x) contains supplementary material, which is available to authorized users.

## Introduction

Enamides are found as structural elements in biologically relevant natural compounds and constitute useful intermediates in organic synthesis [1–4]. Accordingly, the synthesis of enamides has been the subject of considerable investigation in the past decades. However, the preparation of cyclic enamides remains rare [6], in particular when medium-ring derivatives are targeted [5, 6].

**Fig. 1:**
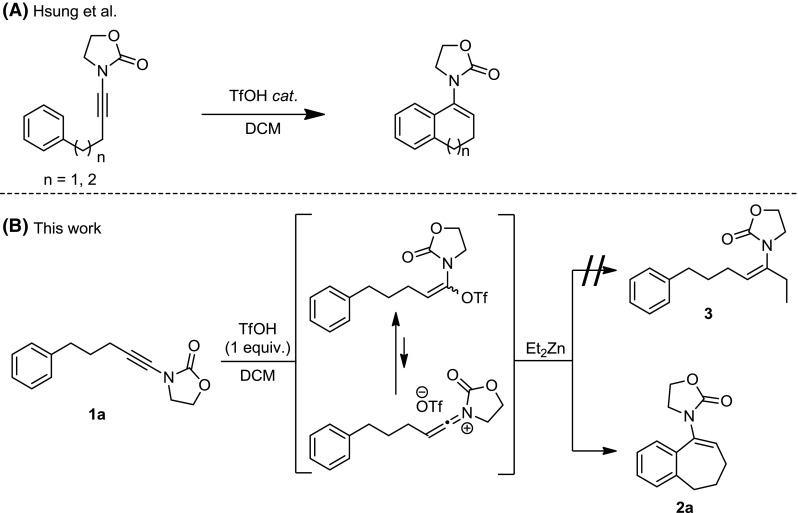
**a** Previous work on ynamide cationic cyclisations and **b** planned synthesis of compound **3** according to our prior report and unexpected observation

As part of our research, we have made extensive use of ynamides as versatile reagents for a range of electrophile-triggered transformations [7–18]. Recently, we developed an efficient approach to prepare α,β-disubstituted enamides via the addition of dialkylzinc reagents to a Bronsted acid-activated ynamide (in the form of a keteniminium/enamide triflate intermediate) [18]. During this study, we found that ynamide **1a**, carrying a phenyl substituent at the end of a three-carbon chain, did not afford the desired product **3** (Fig. 1b). In pioneering work on ynamide cyclizations, Hsung et al. reported an efficient acid-catalyzed process that delivers five- or six-membered ring enamide products as shown in Fig. 1a [6, 19–25]. Surprisingly, the formation of seven-membered ring enamides was not included in that report, which motivated us to study this reaction in more detail. Herein, we wish to report our observations in this study and our understanding of the reaction mechanism.

## Results and discussion

At the outset, we focused on the readily available ynamide **1a** to screen a variety of reaction conditions. When compound **1a** is treated with a full equivalent (1.0 equiv.) of TfOH in DCM, the desired cyclization product is formed in good chemical yield after only 5 min (Table 1, entry 1). Aiming to employ catalytic amounts of TfOH we found that, when 5–10 mol% (0.05–0.1 equiv.) TfOH was used, the yield of product **2a** dropped precipitously (entries 2–5). A consistent trend was observed for even slightly larger amounts of TfOH all the way up to 50 mol% (0.5 equiv.), where no more than 45% of product could be detected (entries 6–10).

**Table 1:** Optimization of reaction conditions
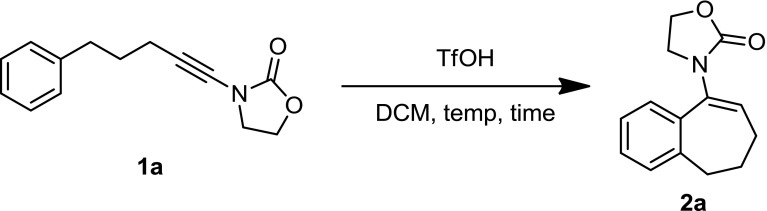

Entry	Time	Temp/°C	Acid	Yield/%
1	5 min	0	1.0 eq TfOH	66^a^
2	2 h	0	5 mol% TfOH	< 5^a^
3	5 h	0	10 mol% TfOH	10^b^
4	5 h	r.t.	10 mol% TfOH	13^b^
5	5 h	50	10 mol% TfOH	14^b^
6	20 h	0^c^	20 mol% TfOH	< 5^b^
7	17 h	0^c^	30 mol% TfOH	7^b^
8	1 h	0	40 mol% TfOH	23^b^
9	5 h	0	50 mol% TfOH	38^b^
10	3 h	0	50 mol% TfOH	45^b^
11	2 h	0	5 mol% Tf_2_NH	6^b^
12	5 h	0	10 mol% Tf_2_NH	7^b^
13	0.5 h	0	1.0 eq TfOH	70^d^
14	1 h	0	1.0 eq TfOH	81^d, e^

^a^Crude NMR

^b^Crude NMR using mesitylene as an internal standard

^c^0 °C to rt over 16 h

^d^Isolated yield

^e^Highest isolated yield

The use of Tf_2_NH did not lead to better results (entries 11, 12) and we eventually acknowledged the need for a full equivalent of TfOH to achieve full conversion. Under those conditions (entry 14), a reaction time of 1 h proved ideal and allowed the obtention of 81% isolated yield of **1a**.

With suitable reaction conditions in hand, a number of ynamides were prepared and screened (Table 2). A *para*-Me substrate **1b** was also a suitable precursor for this cyclization. Disappointingly, the higher homologs **1c** and **1d** did not lead to the products of cyclization (8- and 9-membered ring products **2c**/**2d**). In these cases, only starting material was recovered.

**Table 2:**
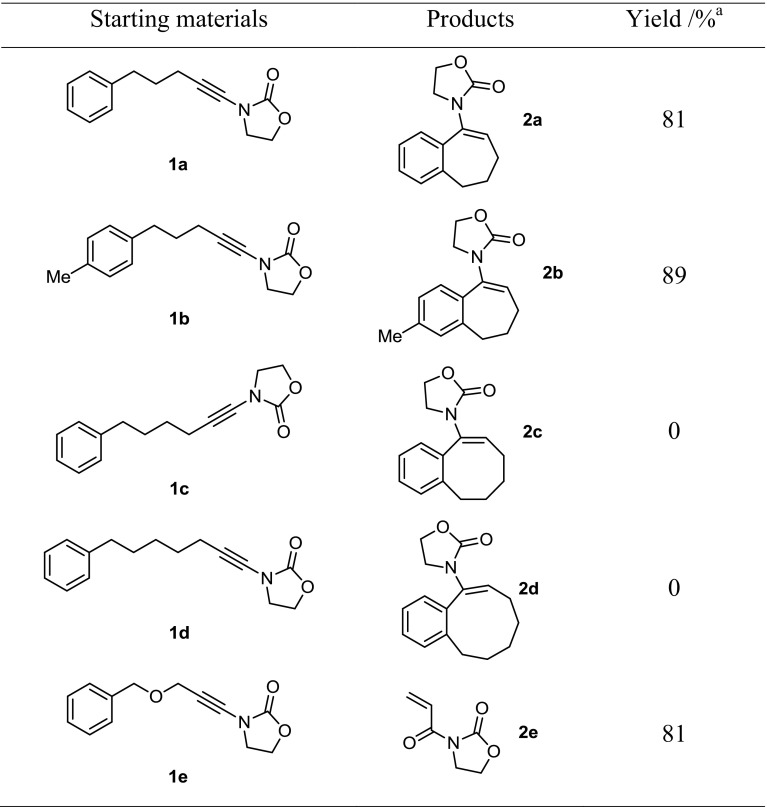
Scope of ynamide cyclization

^a^TfOH (1 equiv.) was added to ynamide **1** in DCM (0.1 M) at 0 °C

**Fig. 2:**
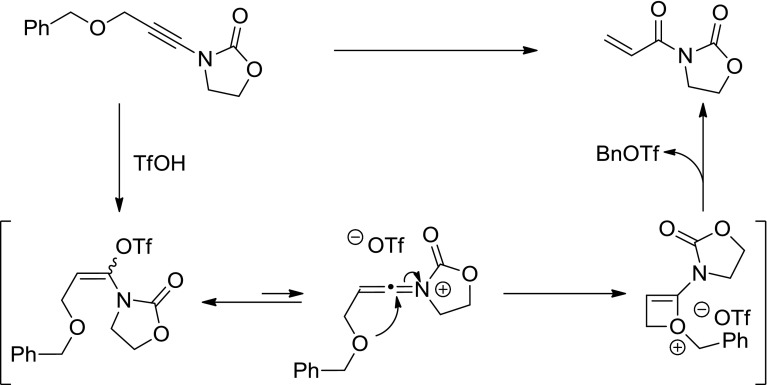
Proposed mechanism for the fragmentation of **2e**

Interestingly, the use of an oxygen-tethered substrate **1e** led cleanly to the formation of acryloyl imide **2e** in 81% yield. We believe that this is the result of a fragmentation process, as outlined in Fig. 2.

The experiments described herein, in particular those in Table 1, reinforce the notion that stoichiometric amounts of TfOH are needed in order to obtain the enamide product. This is at odds with the theoretical mechanism for this cyclization (Fig. 1b), whereby the Friedel–Crafts-like attack of the aromatic onto the keteniminium presupposes transient loss of aromaticity which is later regained by loss of a proton. Reevaluation of the data presented in Table 1 also shows a direct proportionality between conversion/yield of product and catalyst loading, clearly suggesting that catalyst inhibition beyond a single turnover is taking place [19].

**Fig. 3:**
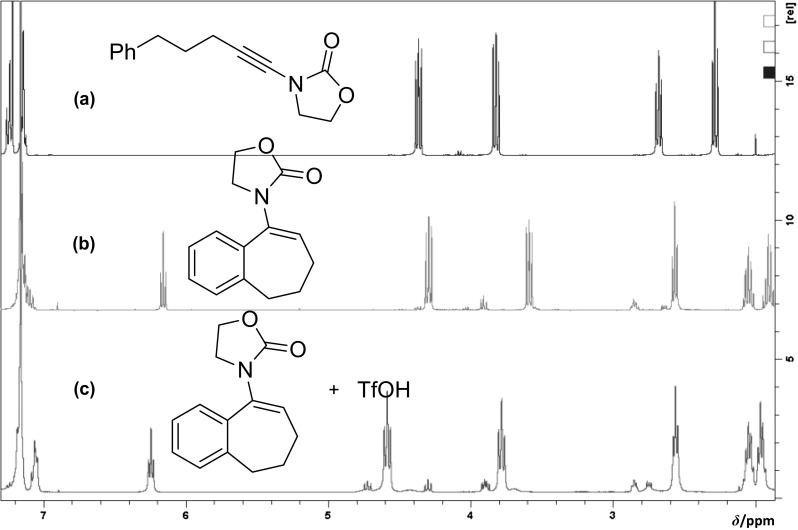
^1^H NMR (400 MHz, CDCl_3_) spectra for: **a** ynamide **1a**; **b** enamide **2a** obtained 15 min after the addition of TfOH; and **c** enamide **2a** after being treated with TfOH for 5 min

To obtain further insight on this, ^1^H NMR studies were carried out in Fig. 3. When **1a** was treated with TfOH in DCM, formation of the desired cyclization product **2a** was observed swiftly (within 15 min). However, upon extension of the reaction time, the product **2a** was slowly converted to another compound. We assigned this new compound as iminium **4a**, the product of protonation of the enamide double bond.

**Fig. 4:**
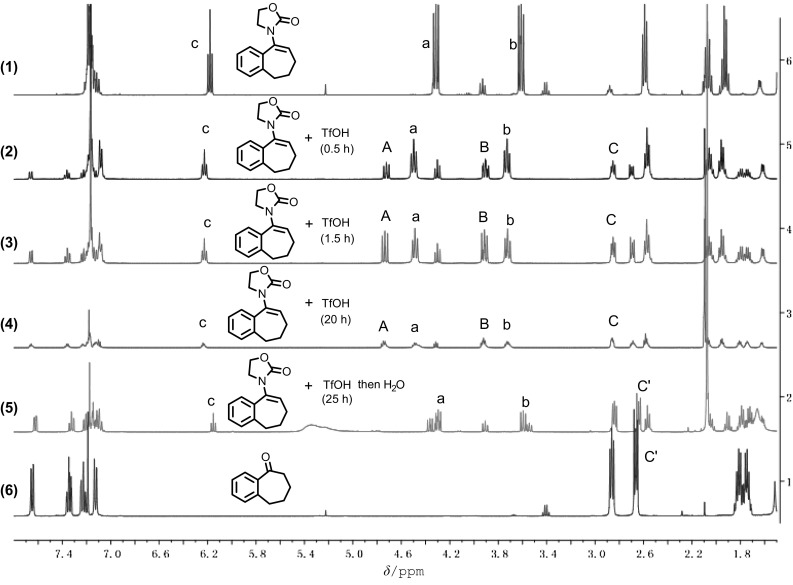
^1^H NMR (400 MHz, CDCl_3_) spectra for: (1) enamide **2a**; (2) enamide **2a** was treated with TfOH for 0.5 h; (3) enamide **2a** was treated with TfOH for 1.5 h; (4) enamide **2a** was treated with TfOH for 20 h; (5) enamide **2a** was treated with TfOH for 25 h, then add H_2_O; and (6) ketone **5**

As shown in Fig. 4, when pure enamide was treated with TfOH for 5 min, the formation of compound **4** was also observed by NMR (spectra 1, 2, and 3). After quenching with water, we can observe the formation of ketone (spectra 4 and 5).

**Fig. 5:**
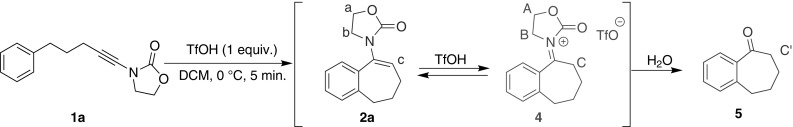
Rationale for the requirement for stoichiometric amounts of TfOH

Taking all this information into account (Fig. 5), it becomes apparent when catalytic amounts of TfOH were used, the initially formed **2a** further reacts with TfOH to form compound **4a**. This ultimately results in consumption of all available TfOH. With no addditional acidic catalyst to promote the cyclization of **1a**, conversion of **2a** then naturally stopped at a level of actual loading of TfOH.

## Conclusion

We have reported herein a Brønsted acid-catalyzed arene-ynamide cyclization for the formation of a seven-membered ring enamide. Through mechanistic studies, an unusual product inhibition behavior was observed which mandated the use of stoichiometric amounts of TfOH.

## Experimental

All glassware was dried before use. All solvents were used in p.a. quality. All reagents were used as received from commercial suppliers unless otherwise stated. Reaction progress was monitored using TLC on aluminum sheets coated with silica gel 60 with 0.2 mm thickness (Pre-coated TLC-sheets ALUGRAM^**®**^ Xtra SIL G/UV254). Visualization was achieved by UV light (254 nm and 363 nm) and/or by treatment with potassium manganite(VII) and heat. CC was performed using silica gel 60 (230–400 Mesh, MERCK AND CO.). All ^1^H NMR and ^13^C NMR spectra were recorded on BRUKER AVIII-400 in CDCl_3_. Chemical shifts (*δ*) were given in “parts per million” (ppm), referenced to the peak of TMS (*δ *= 0.00 ppm), using the solvent as internal standard (^1^H: *δ*(CDCl_3_) = 7.26 ppm; ^13^C: *δ*(CDCl_3_) = 77.16 ppm [26]). Coupling constants (*J*) were given in Hz. Spectroscopy splitting patterns were designated as singlet (s), doublet (d), triplet (t), quartet (q), pentet (p), multiplet (m), or combinations of that. MS were obtained using a BRUKER maXis spectrometer with ESI and the main signals were given in *m*/*z* units. IR were recorded on a BRUKER VERTEX FT-IR spectrometer. The following computer programs were used: MestReNova from Mestrelab Research and ChemDraw from PerkinElmer.

### General procedure of synthesis of ynamides

In a 250 cm^3^ two-neck round-bottom flask equipped with a stir-bar, CuCl_2_ (0.2 equiv.), nitrogen nucleophile (5 equiv.), and Na_2_CO_3_ (2 equiv.) were combined. The reaction flask was purged with oxygen gas. A solution of pyridine (2 equiv.) in dry toluene (0.06 M) was added to the reaction flask and stirred at 70 °C. After 0.5 h, a solution of the respective alkyne (1.0 equiv.) in dry toluene (0.033 M) was added to the flask over 4 h using a syringe pump. After addition of alkyne/toluene solution, the reaction mixture was allowed to stir at 70 °C overnight. After cooling to r.t., the crude mixture was concentrated under reduced pressure. The residue was purified by column chromatography on silica gel.

#### 3-(5-Phenylpent-1-yn-1-yl)oxazolidin-2-one (**1a**, C_14_H_15_NO_2_)

According to the general procedure, the ynamide **1a** was isolated after purification by column chromatography (pentane/EE = 5:1–1:1) as a yellow oil (1555 mg, 6.8 mmol, 68%). ^1^H NMR (400 MHz, CDCl_3_): *δ* = 7.30 (t, 2H), 7.21 (dd, *J* = 9.9, 4.1 Hz, 3H), 4.43 (t, 2H), 3.89 (t, 1H), 2.75 (m, 2H), 2.35 (t, *J* = 7.1 Hz, 2H), 1.88 (p, 2H) ppm; ^13^C NMR (100 MHz, CDCl_3_): *δ* = 141.6, 128.7, 128.5, 126.0, 70.9, 62.9, 47.2, 34.9, 30.4, 18.0 ppm; IR: $\bar{\nu }$ = 3060, 3026, 2924, 2861, 2270, 1766, 1603, 1479, 1454, 1415, 1302, 1207, 1113, 1035, 972, 748, 701 cm^−1^; HRMS (ESI): *m*/*z* calculated for [M]^+^ 229.1103, found 229.1093.

#### 3-(5-Phenylpent-1-yn-1-yl)oxazolidin-2-one (**1b**, C_15_H_17_NO_2_)

According to the general procedure, **1b** was isolated after purification by column chromatography [pentane/EE = 5:1–1:1] as orange crystals (0.71 g, 2.9 mmol, 33%). ^1^H NMR (600 MHz, CDCl_3_): *δ* = 7.11–7.06 (m, 4H), 4.43–4.39 (m, 2H), 3.89–3.85 (m, 2H), 2.68 (t, 2H), 2.34–2.29 (m, 5H), 1.87–1.80 (m, 2H) ppm; ^13^C NMR (151 MHz, CDCl_3_): *δ* = 156.7, 138.6, 135.5, 129.2, 128.5, 71.0, 70.6, 62.9, 47.2, 34.5, 30.6, 21.1, 18.0 ppm; IR: $\bar{\nu }$ = 2923, 2860, 2271, 1767, 1515, 1479, 1415, 1302, 1206, 1112, 1036, 806, 750 cm^−1^; HRMS (ESI): *m*/*z* calculated for [M+Na]^+^ 266.1157, found 266.1153.

#### 3-(6-Phenylhex-1-yn-1-yl)oxazolidin-2-one (**1c**, C_15_H_17_NO_2_)

According to the general procedure, **1c** was isolated after purification by column chromatography (pentane/EE = 5:1–1:1) as a yellow oil (43 mg, 0.19 mmol, 9%). Starting material was recovered as a yellow oil (211 mg, 1.3 mmol, 67%). ^1^H NMR (400 MHz, CDCl_3_): *δ* = 7.28 (ddd, *J* = 7.2, 3.5, 1.4 Hz, 2H), 7.17 (m, 3H), 4.40 (dt, 2H), 3.85 (dt, 2H), 2.63 (t, *J* = 7.6 Hz, 2H), 2.34 (t, *J* = 7.1 Hz, 2H), 1.73 (m, 2H), 1.57 (m, 2H) ppm; ^13^C NMR (100 MHz, CDCl_3_): *δ* = 156.8, 142.4, 128.6, 128.4, 125.9, 71.1, 70.3, 62.9, 60.6, 47.2, 35.5, 31.1, 30.7, 28.4, 18.5 ppm; IR: $\bar{\nu }$ = 2925, 2855, 2270, 1602, 1479, 1454, 1414, 1302, 1204, 1112, 1035, 973, 748, 700, 618 cm^−1^; HRMS (ESI): *m/z* calculated for [M]^+^ 243.1259, found 243.1252.

#### 3-(7-Phenylhept-1-yn-1-yl)oxazolidin-2-one (**1d**, C_16_H_19_NO_2_)

According to the general procedure, **1d** was isolated after purification by column chromatography (pentane/EE = 5:1–1:1) as a yellow oil (197 mg, 0.77 mmol, 44%). Starting material was recovered as a yellow oil (136 mg, 0.79 mmol, 45%). ^1^H NMR (400 MHz, CDCl_3_): *δ* = 7.27 (dd, *J* = 10.6, 5.7 Hz, 3H), 7.17(m, 2H), 4.40 (dd, *J* = 8.6, 7.4 Hz, 2H), 3.84 (dd, *J* = 8.6, 7.4 Hz, 2H), 2.62 (t, 2H), 2.30 (t, *J* = 7.2 Hz, 2H), 1.63 (dt, *J* = 15.4, 7.7 Hz, 2H), 1.56 (dt, 2H), 1.43 (m, 2H) ppm; ^13^C NMR (100 MHz, CDCl_3_): *δ* = 156.8, 142.7, 128.6, 128.4, 125.8, 71.3, 70.3, 62.9, 47.2, 35.9, 31.1, 28.8, 28.6, 18.5 ppm; IR: $\bar{\nu }$ = 3060, 3025, 2931, 2857, 2266, 1769, 1603, 1480, 1454, 1415, 1302, 1264, 1206, 1113, 1036, 747, 701 cm^−1^; HRMS (ESI): *m/z* calculated for [M]^+^ 257.1416, found 257.1412.

### General procedure of cyclization of ynamide

The respective ynamide (0.20 mmol, 1.0 equiv.) was dissolved in dry DCM under inert atmosphere at 0 °C. Triflic acid (0.20 mmol, 1.0 equiv.) was added dropwise using a microsyringe and stirred for 1 h. The reaction was quenched by the addition of a sat. NH_4_Cl solution. The reaction mixture was extracted with DCM and the combined organic phases were dried over MgSO_4_ and concentrated under reduced pressure. The residue was purified by column chromatography on silica gel.

#### 3-(6,7-Dihydro-5*H*-benzo[*g*]annulen-9-yl)oxazolidin-2-one (**2a**, C_14_H_15_NO_2_)

According to the general procedure, 69 mg **1a** (0.30 mmol, 1.0 equiv.) and 45 mg triflic acid (0.30 mmol, 1.0 equiv.) were reacted in 3 cm^3^ DCM and the corresponding cyclization product **2a** was isolated after purification by column chromatography (pentane/EE = 3:1–1:1) as a white solid (56 mg, 0.24 mmol, 81%). The ketone side product was isolated as white solid (7 mg, 0.03 mmol, 10%). ^1^H NMR (400 MHz, CDCl_3_): *δ* = 7.24 (m, 5H), 6.25 (t, *J* = 7.1 Hz, 1H), 4.39 (ddd, *J* = 8.1, 7.2, 2.7 Hz, 2H), 3.68 (t, 2H), 2.66 (t, *J* = 6.8 Hz, 2H), 2.15 (p, 2H), 2.00 (p, 2H) ppm; ^13^C NMR (100 MHz, CDCl_3_): *δ* = 129.5, 128.2, 126.6, 126.4, 124.3, 61.8, 46.7, 34.7, 32.6, 24.1 ppm; IR: $\bar{\nu }$ = 2928, 2857, 1751, 1634, 1481, 1452, 1112, 1086, 1038, 898, 773, 756, 730, 702, 607 cm^−1^; HRMS (ESI): *m/z* calculated for: [M]^+^ 229.1103, found 229.1094.

#### 3-(3-Methyl-6,7-dihydro-5*H*-benzo [7] annulen-9-yl)oxazolidin-2-one (**2b**, C_15_H_17_NO_2_)

According to the general procedure, **2b** was isolated as a white solid (109 mg, 0.446 mmol, 90%). The ketone side product **8** was isolated as a colorless oil (0.8 mg, 0.005 mmol, 1%). ^1^H NMR (600 MHz, CDCl_3_): *δ* = 7.14–7.00 (m, 4H), 6.23 (t, *J* = 7.2 Hz, 1H), 4.41–4.38 (m, 2H), 3.70–3.65 (m, 2H), 2), 2.61 (t, *J* = 6.9 Hz, 2H), 2.33 (s, 3H), 2.11 (p, *J* = 7.2 Hz, 2H), 1.98 (q, *J* = 7.2 Hz, 2H) ppm; ^13^C NMR (151 MHz, CDCl_3_): *δ* = 156.9, 139.3, 135.8, 135.4, 134.8, 130.7, 129.4, 129.0, 127.0, 124.4 ppm; IR: $\bar{\nu }$ = 2923, 2855, 1743, 1632, 1480, 1447, 1397, 1364, 1287, 1258, 1221, 1147, 1119, 1083, 1066, 1036, 976, 923, 825, 794, 757, 732, 683 cm^−1^; HRMS (ESI): *m/z* calculated for [M+H]^+^ 244.1338, found 244.1335.

#### 3-Acryloyloxazolidin-2-one (**2e**, C_6_H_7_NO_3_)

According to the general procedure, 46 mg **1e** (0.2 mmol, 1.0 equiv.) and 30 mg triflic acid (0.2 mmol, 1.0 equiv.) were reacted in 2 cm^3^ DCM and the amide **2e** was isolated after purification by column chromatography (pentane/EE = 3:1–1:1) as a white solid (25 mg, 0.18 mmol, 89%). ^1^H NMR (400 MHz, CDCl_3_): *δ* = 7.50 (dd, 1H), 6.56 (dd, *J* = 17.0, 1.8 Hz, 1H), 5.90 (dd, *J* = 10.5, 1.8 Hz, 1H), 4.44 (t, 2H), 4.09 (t, 2H) ppm; ^13^C NMR (100 MHz, CDCl_3_): *δ* = 165.2, 153.5, 131.9, 127.1, 62.3, 42.8 ppm; IR: $\bar{\nu }$ = 1775, 1717, 1687, 1412, 1389, 1327, 1264, 1118, 1066, 1040, 1014, 732, 702 cm^−1^; HRMS (ESI): *m/z* calculated for [M]^+^ 141.0426, found 141.0417.

## Electronic supplementary material

Below is the link to the electronic supplementary material.

Supplementary material 1 (PDF 360 kb)
