# Supplementary material for: On the formation of seven-membered rings by arene-ynamide cyclization
Source: Monatsh Chem. 2018 Nov 16;150(1):3–10. doi: 10.1007/s00706-018-2320-x (PMC6320749; doi:10.1007/s00706-018-2320-x)
Supplement: Supplementary file 1 — Supplementary material 1 (PDF 360 kb) [file 706_2018_2320_MOESM1_ESM.pdf]

On the formation of seven-membered rings by arene-ynamide cyclisation

Bogdan R. Brutiu, W. Andrei Bubeneck, Olivera Cvetkovic, Jing Li and Nuno Maulide*

Table of Contents

General Information	Page 1
Preparation of Starting materials and characterization of products	Page 1
NMR Spectra	Page 8

1. General Information

Unless otherwise stated, All glassware was dried before use. All solvents were used in p.a. quality. All reagents were used as received from commercial suppliers unless otherwise stated. Reaction progress was monitored using TLC on aluminum sheets coated with silica gel 60 with 0.2mm thickness (Pre-coated TLC-sheets ALUGRAM® Xtra SIL G/UV254). Visualization was achieved by UV light (254 nm and 363 nm) and/or by treatment with potassium manganite (VII) and heat. CC was performed using silica gel 60 (230-400 Mesh, MERCK AND CO.). All ^{1}H-NMR, ^{13}C-NMR, spectra were recorded on BRUKER AVIII400 in CDCl3. Chemical shifts (*δ*) were given in "parts per million" (ppm), referenced to the peak of TMS (*δ* = 0.00 ppm), using the solvent as internal standard (^{1}H: $\delta(CDCl_3)$ = 7.26 ppm; ^{13}C: $\delta(CDCl_3)$ = 77.16 ppm).[19] Coupling constants (*J*) were given in Hz. Spectroscopy splitting patterns were designated as singlet (s), doublet (d), triplet (t), quartet (q), pentet (p) multiplet (m) or combinations of that. MS were obtained using a BRUKER maXis spectrometer with ESI and the main signals were given in *m/z* units. IR were recorded on a BRUKER VERTEX FT-IR spectrometer. The following computer programs were used: MestReNova from Mestrelab Research and ChemDraw from PerkinElmer.

2 Preparation of Starting Material

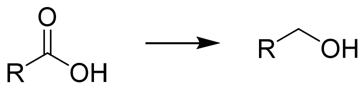

GP1: The respective acid (10.0 mmol, 1.0 equiv.) was dissolved in dry THF at rt in a 250 ml open round bottom flask equipped with a stir-bar. One pellet of $LiAlH_4$ (20.0 mmol, 2.0 equiv.) was added to the reaction mixture and stirred for 4 h at rt and then overnight at 50 °C under reflux. The reaction was quenched by addition of a sat. potasium sodium tartrate solution. The organic phase has been decanted and the solid residue washed with Et_2O. The combined organic phases were dried over $MgS0_4$ and the solvent removed under reduced pressure to obtain the corresponding alcohol. The compound was used in the next step without further purification.

5-Phenylpentan-1-ol

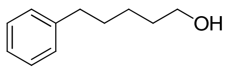

According to **GP1**, 5-phenylvaleric acid (1782 mg, 10.00 mmol, 1.0 equiv.) and $LiAlH_4$ (759 mg, 20.00 mmol, 2.0 equiv.) were reacted in THF (75 ml) and the 5-phenylpentan-1-ol was obtained as a pale yellow oil (1624 mg, 8.90 mmol, 89%). **^{1}H-NMR** (400 MHz, $CDCl_3$): δ = 7.26 (dd, *J* = 14.1, 6.4 Hz, 2H), 7.16 (t, 3H), 3.62 (t, *J* = 6.6 Hz, 2H), 2.61 (t, 2H), 1.64 (dt, *J* = 15.5, 7.7 Hz, 2H), 1.58 (p, 2H), 1.41 (p, 2H); **^{13}C-NMR** (100 MHz, $CDCl_3$): δ = 142.7, 128.5, 128.4, 125.8, 63.1, 36.0, 32.8, 31.4, 25.5; **HRMS-ESI** calcd. for: $C_{11}H_{16}O$: 164.1196, found: 164.1191 ([M]+); **IR**: 3348, 3085, 3063, 3027, 2932, 2858, 1603, 1495, 1454, 1054, 1012, 746, 699 cm^{-1}.

3-(p-tolyl)propan-1-ol

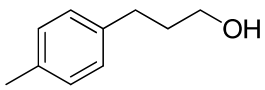

According to **GP1**, the 3-(p-tolyl)propan-1-ol was obtained as a pale yellow oil (1624 mg, 8.90 mmol, 89%). oil (1.93 g, 12.9mmol, 64.4%). **^{1}H-NMR (400MHz, CDCl3):** δ = 7.14–7.07 (m, 4H), 3.67 (t, *J* = 6.4Hz, 2H), 2.71–2.64 (m, 2H), 2.33 (s, 3H), 1.93–1.83 (m, 3H). **TLC:** *R*f = 0.29 (Heptane/ EtOAc = 3/2) [UV].

^{1}H-NMR spectrum is consistent with reported result.

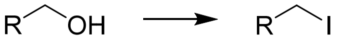

GP2: The respective alcohol (10.0 mmol, 1.0 equiv.), I_2 (11.0 mmol, 1.1 equiv.) and imidazole (20.0 mmol, 2.0 equiv.) were dissolved in 40 ml dry DCM and stirred at rt. After 5 min, a solution of triphenylphosphine (10.5 mmol, 1.05 equiv.) was added and the resulting mixture was stirred for 1 h. The reaction was quenched by addition of a sat. $Na_2S_2O_3$ solution. The reaction mixture was extracted with DCM, the combined organic phases were dried over $MgSO_4$ and the reaction mixture reduced to 20 ml. After addition of 100 ml Pentane, the reaction mixture was reduced to 10 ml, filtered, washed with Pentane and concentrated under reduced pressure. The residue was purified by column chromatography on silica gel.

(4-Iodobutyl)benzene

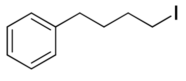

According to **GP2**, (4-Iodobutyl)benzene (1502 mg, 10.0 mmol, 1.0 equiv.), I_2 (2792 mg, 11.0 mmol, 1.1 equiv.), imidazole (1362 mg, 20.0 mmol, 2.0 equiv.) and triphenylphosphine (2754 mg, 10.5 mmol, 1.05 equiv.) were reacted in DCM (50 ml) and the corresponding alkyne iodide (4-Iodobutyl)benzene was isolated after purification by column chromatography (Pentane/EE = 5:1) as a pale yellow oil (2092 mg, 8.04 mmol, 80%). **^{1}H-NMR** (400 MHz, $CDCl_3$): δ = 7.29 (t, *J* = 7.6 Hz, 2H), 7.19 (dd, *J* = 14.3, 7.2 Hz, 3H), 3.20 (t, *J* = 7.0 Hz, 2H), 2.64 (t, *J* = 7.7 Hz, 2H), 1.86 (p, 2H), 1.74 (dq, *J* = 15.0, 7.5 Hz, 2H); **^{13}C-NMR** (100 MHz, $CDCl_3$): δ = 141.9, 128.5, 126.0, 34.9, 33.1, 32.3, 31.1, 6.9; **HRMS-ESI** calcd. for: $C_9H_{11}I$: 260.0056, found: 260.0051 ([M]+); **IR**: 3083, 3061, 3025, 2932, 2855, 1603, 1495, 1453, 1427, 1352, 1251, 1225, 1206, 1167, 1117, 1072, 1030, 908, 744, 698 cm^{-1}.

(5-Iodopentyl)benzene

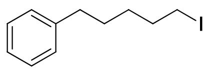

According to **GP2**, (5-Iodopentyl)benzene (1363 mg, 8.3 mmol, 1.0 equiv.), I_2 (2317 mg, 9.1 mmol, 1.1 equiv.), imidazole (1130 mg, 16.6 mmol, 2.0 equiv.) and triphenylphosphine (2286 mg, 8.7 mmol, 1.05 equiv.) were reacted in DCM (42 ml) and the corresponding alkyle iodide (5-Iodopentyl)benzene was isolated after purification by column chromatography (Pentane/EE = 5:1) as a pale yellow oil (1791 mg, 6.5 mmol, 79%). **^{1}H-NMR** (400 MHz, $CDCl_3$): δ = 7.28 (t, *J* = 7.6 Hz, 2H), 7.19 (t, *J* = 8.9 Hz, 3H), 3.19 (t, *J* = 7.0 Hz, 2H), 2.63 (t, 2H), 1.86 (p, 2H), 1.65 (p, 2H), 1.45 (p, 2H); **^{13}C-NMR** (100 MHz, $CDCl_3$): δ = 142.4, 128.5, 128.5, 125.9, 35.9, 33.6, 30.5, 30.3, 7.1; **HRMS-ESI** calcd. for: $C_{10}H_{13}I$: 274.0213, found: 274.0212 ([M]+); **IR**: 3083, 3060, 3024, 2928, 2853, 1943, 1871, 1803, 1740, 1602, 1494, 1452, 1426, 1198, 1164, 1078, 1029, 907, 842, 792, 745, 696, 606 cm^{-1}.

1-(3-iodopropyl)-4-methylbenzene

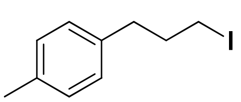

According to **GP2**, 1-(3-iodopropyl)-4-methylbenzene was isolated as a yellow oil (2.63 g, 10.1mmol, 79%). **^{1}H-NMR** (600MHz, $CDCl_3$): δ = 7.12–7.07 (m, 1H), 3.17 (t, *J* = 6.8Hz, 2H), 2.69 (t, *J* = 7.3Hz, 2H), 2.32 (s, 1H), 2.14–2.08 (m, 2H). **^{13}C-**

NMR (151MHz, CDCl3): ± (ppm) = 137.5, 135.8, 129.3, 128.6, 35.9, 35.2, 21.2, 6.6. **HRMS (EI):** found: 260.0053 ([M]+), calcd. for C10H13I: 260.0062. **IR:** 3007, 2921, 2653, 1514, 1446, 1424, 1276, 1261, 1210, 1165, 802, 766, 755, 746 cm^{-1}.

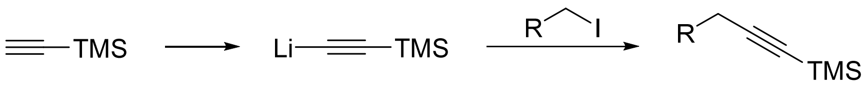

GP3: To a flame dried 100 mL round bottomed flask, trimethyl(prop-1-ynyl)silane (16.0 mmol, 1.6 equiv.) was dissolved in 50 ml dry THF. The reaction flask was cooled to –30 °C and *n*-BuLi (15.0 mmol, 6 mL, 2.5 M solution in hexanes, 1.5 equiv.) was added dropwise. After 1 h the respective alkyle iodide (10 mmol, 1.0 equiv.) was added drop wise and the solution was allowed to warm to rt over 4 h and then stirred overnight at 50 °C under reflux. The reaction was quenched by addition of a sat NH_4Cl solution. The reaction mixture was extracted with Et_2O and the solvent removed under reduced pressure. The residue was purified by column chromatography on silica gel.

Trimethyl(6-phenylhex-1-yn-1-yl)silane

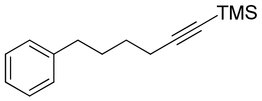

According to **GP3**, Trimethyl(6-phenylhex-1-yn-1-yl)silane (1178 mg, 4.5 mmol, 1.0 equiv.), trimethyl(prop-1-ynyl)silane (712 mg, 7.2 mmol, 1.6 equiv.) and *n*-BuLi (6.8 mmol, 2.7 mL, 2.5 M solution in hexanes, 1.5 equiv.) were reacted in THF (23 ml) and the Trimethyl(6-phenylhex-1-yn-1-yl)silane was isolated after purification by column chromatography (Pentane) as a colorless oil (985 mg, 4.3 mmol, 94%). **^{1}H-NMR** (400 MHz, $CDCl_3$): δ = 7.28 (dd, *J* = 9.8, 5.4 Hz, 2H), 7.18 (m, 3H), 2.63 (t, *J* = 7.7 Hz, 2H), 2.25 (t, *J* = 7.2 Hz, 2H), 1.74 (p, 2H), 1.57 (h, *J* = 4.1 Hz, 2H), 0.15 (s, 9H); **^{13}C-NMR** (100 MHz, $CDCl_3$): δ = 142.5, 128.6, 128.4, 125.9, 107.5, 84.7, 35.5, 30.6, 28.2, 19.9, 18.4, 0.3; **HRMS (ESI)** calcd. for: $C_{15}H_{22}Si$: 230.1485, found: 230.1483 ([M]+); **IR**: 3063, 3027, 2937, 2859, 2173, 1604, 1496, 1454, 1428, 1326, 1248, 1067, 1049, 1030, 981, 936, 838, 758, 698, 640 cm^{-1}.

Trimethyl(7-phenylhept-1-yn-1-yl)silane

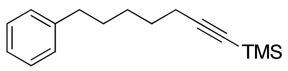

According to **GP3**, Trimethyl(7-phenylhept-1-yn-1-yl)silane (1778 mg, 6.5 mmol, 1.0 equiv.), trimethyl(prop-1-ynyl)silane (1019 mg, 10.4 mmol, 1.6 equiv.) and *n*-BuLi (9.7 mmol, 3.9 mL, 2.5 M solution in hexanes, 1.5 equiv.) were reacted in THF (33 ml) and the trimethyl(7-phenylhept-1-yn-1-yl)silane was isolated after purification by column chromatography (Pentane) as a yellow oil (1281 mg, 5.2 mmol, 81%). **^{1}H-NMR** (400 MHz, $CDCl_3$): δ = 7.13 (dd, *J* = 10.0, 4.8 Hz, 2H), 7.02 (dd, *J* = 9.4, 3.9 Hz, 3H), 2.47 (t, 2H), 2.08 (t, *J* = 7.1 Hz, 2H), 1.50 (dt, *J* = 15.3, 7.5 Hz, 2H), 1.41 (dt, *J* = 14.1, 7.0 Hz, 2H), 1.29 (ddd, *J* = 12.3, 5.7, 3.1 Hz, 2H), 0.00 (s, 9H); **^{13}C-NMR** (100 MHz, $CDCl_3$): δ = 142.6, 128.4, 128.2, 125.6, 107.5, 84.4, 35.8, 28.4, 28.4, 19.8, 18.3, 0.1; **HRMS (ESI)** calcd. for: $C_{16}H_{24}Si$: 244.1647, found: 244.1632 ([M]+); **IR**: 2934, 2858, 2174, 1604, 1496, 1454, 1249, 1048, 1031, 839, 759, 747, 698, 640 cm^{-1}.

Trimethyl(5-(p-tolyl)pent-1-yn-1-yl)silane

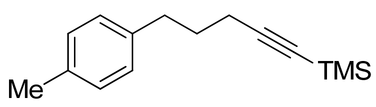

^{1}H-NMR (400MHz, CDCl3): δ = 7.12–7.06 (m, 4H), 2.68 (t, *J* = 7.7Hz, 2H), 2.32 (s, 3 H), 2.23 (t, *J* =7.1Hz, 2H), 1.88–1.77 (m, 2H). The crude product further go to next step withoutpurification.

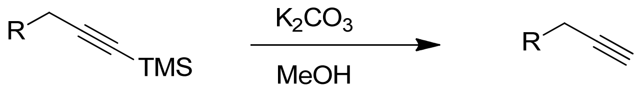

GP4: The respective compound (1 equiv.) and K_2CO_3 (2.0 equiv.) were dissolved in MeOH (ca. 0.3 M) and stirred at rt overnight. The reaction mixture was concentrated under reduced pressure, diluted with Et_2O, washed with sat. NH_4Cl solution, dried over $MgSO_4$ and concentrated under reduced pressure. The residue was purified by column chromatography on silica gel.

Hex-5-yn-1-ylbenzene

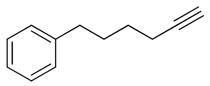

According to **GP4**, Hex-5-yn-1-ylbenzene (956 mg, 4.2 mmol, 1.0 equiv.) and K_2CO_3 (1158 mg, 8.4 mmol, 2.0 equiv.) were reacted in MeOH (14 ml) and the corresponding alkyne Hex-5-yn-1-ylbenzene was isolated after purification by column chromatography (Pentane) as a yellow oil (613 mg, 3.87 mmol, 93.3%). **^{1}H-NMR** (400 MHz, $CDCl_3$): δ = 7.29 (t, 2H), 7.18 (m, 3H), 2.64 (t, 2H), 2.22 (td, J = 7.1, 2.6 Hz, 2H), 1.95 (t, J = 2.6 Hz, 1H), 1.75 (ddd, J = 15.5, 11.2, 7.7, 2H), 1.58 (dt, 2H); **^{13}C-NMR** (100 MHz, $CDCl_3$): δ = 142.4, 128.5, 128.4, 125.9, 84.6, 68.5, 35.5, 30.6, 28.1, 18.4; **HRMS (ESI)** calcd. for: $C_{12}H_{14}$: 158.1090, found: 158.1085 ([M]+); **IR**: ν = 3299, 3063, 3027, 2938, 2860, 1604, 1496, 1454, 1432, 1328, 1084, 1031, 747, 699, 639 cm^{-1}.

Hept-6-yn-1-ylbenzene

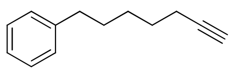

According to **GP4**, Hept-6-yn-1-ylbenzene (531 mg, 2.2 mmol, 1.0 equiv.) and K_2CO_3 (303 mg, 4.4 mmol, 2.0 equiv.) were reacted in MeOH (8 ml) and the corresponding alkyne Hept-6-yn-1-ylbenzene was isolated after purification by column chromatography (Pentane) as a yellow oil (329 mg, 1.9 mmol, 88%). **^{1}H-NMR** (400 MHz, $CDCl_3$): δ = 7.28 (t, *J* = 7.6 Hz, 2H), 7.18 (m, 3H), 2.62 (t, 1H), 2.19 (td, *J* = 7.1, 2.6 Hz, 2H), 1.95 (t, *J* = 2.6 Hz, 1H), 1.64 (dt, *J* = 15.5, 7.7 Hz, 2H), 1.56 (p, 2H), 1.46 (p, 2H); **^{13}C-NMR** (100 MHz, $CDCl_3$): δ = 142.6, 128.4, 128.3, 125.6, 84.6, 68.2, 35.8, 31.0, 28.4, 28.3, 18.34; **HRMS (ESI)** calcd. for: $C_{13}H_{16}$: 172.1247, found: 172.1245 ([M]+); **IR**: 3303, 3062, 3026, 2933, 2857, 2118, 1603, 1496, 1454, 1433, 1264, 1030, 741, 699, 634 cm^{-1}.

1-methyl-4-(pent-4-yn-1-yl)benzene

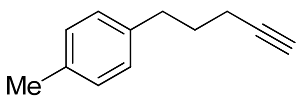

The residue was purified by column chromatography on silica gel (4% EtOAc in Heptane) and 1-methyl-4-(pent-4-yn-1-yl)benzene was isolated as a yellow oil (1.46 g, 9.2mmol, 92.9%). **^{1}H-NMR** (600MHz, CDCl3)**:** δ = 7.12–7.07 (m, 4H), 2.72–2.66 (m, 2H), 2.32 (s, 3H), 2.29–2.17 (m, 2H), 1.99 (t, *J* = 2.6Hz, 1H), 1.86–1.80 (m,

2H). **HRMS (EI):** found: 158.1087 ([M]+), calcd. for C12H14: 158.1096. **IR:** 3007, 2990, 2941, 2859, 1515, 1456, 1276, 1261, 804, 758, 749 cm^{-1}.

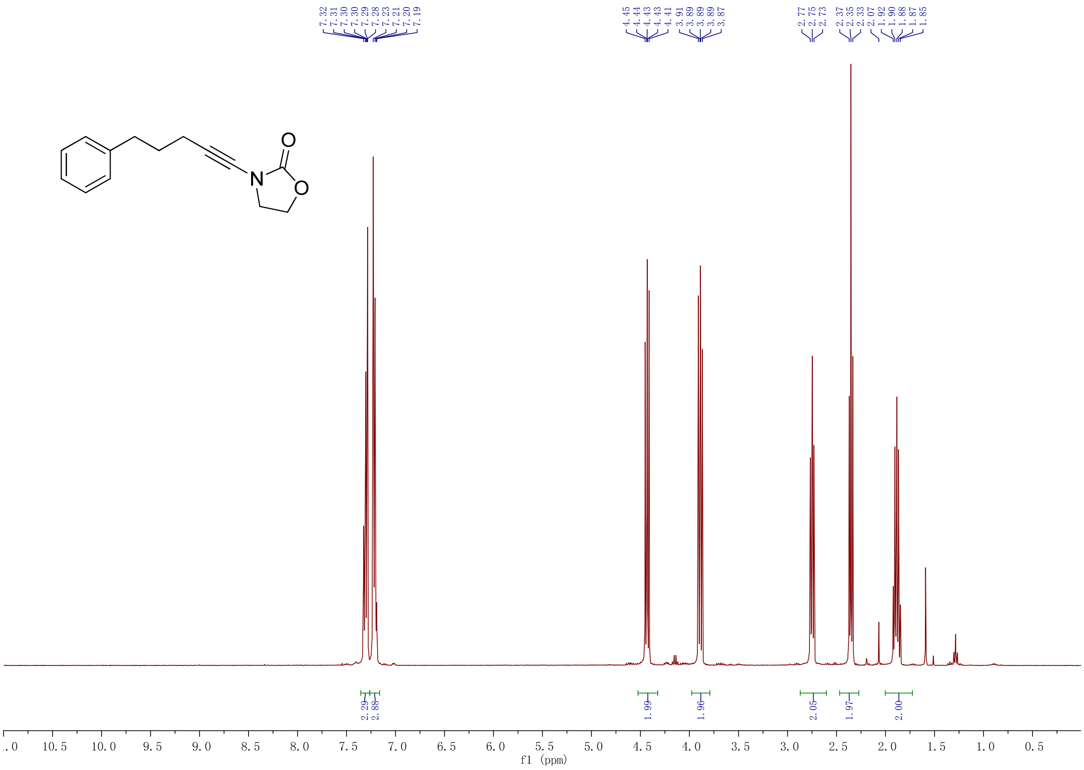
7.32
7.31
7.30
7.30
7.29
7.28
7.23
7.21
7.20
7.19
4.45
4.44
4.43
4.43
4.41
3.91
3.89
3.89
3.87
2.77
2.75
2.73
2.37
2.35
2.33
2.07
1.92
1.90
1.88
1.87
1.85
N
O
O
2.29
2.88
1.99
1.96
2.05
1.97
2.00
f1 (ppm)

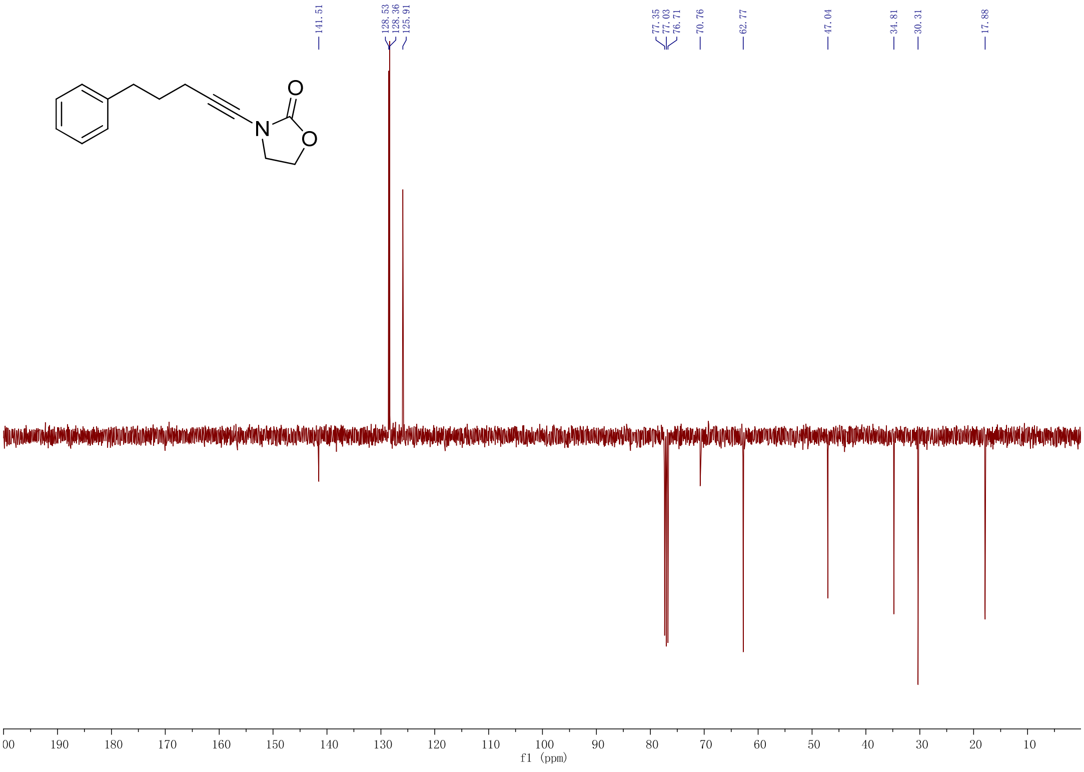
141.51
128.53
128.36
125.91
77.35
77.03
76.71
70.76
62.77
47.04
34.81
30.31
17.88
N
O
O
f1 (ppm)

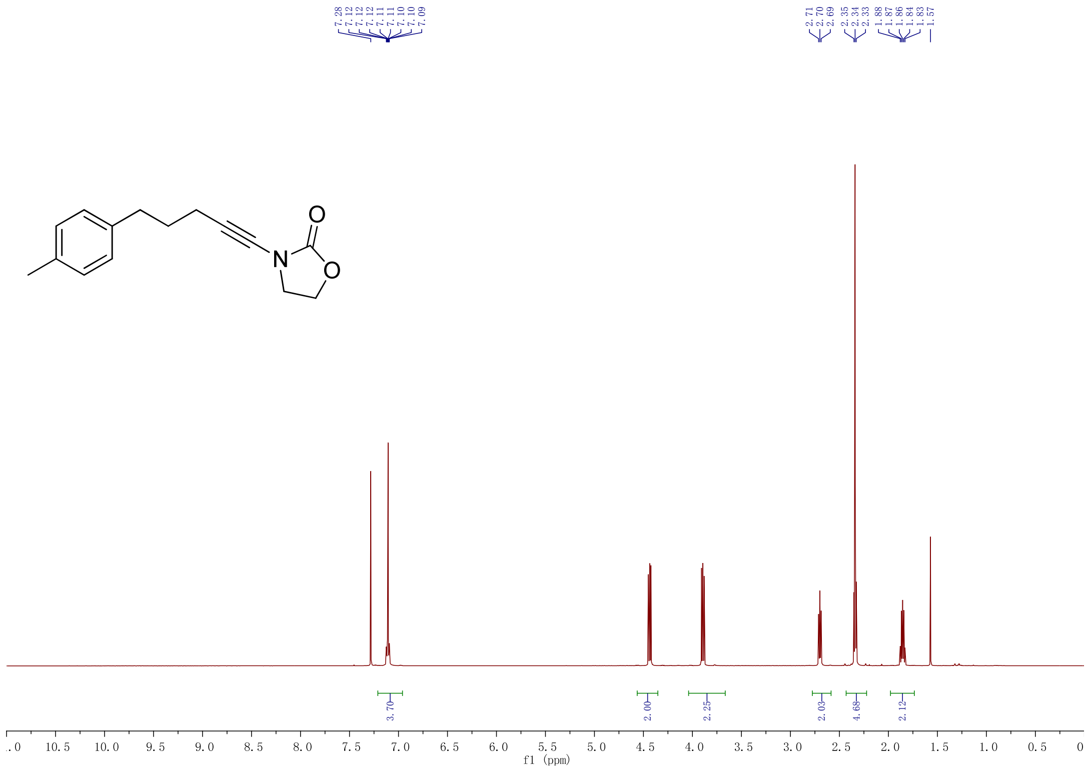
7.28
7.12
7.12
7.12
7.11
7.10
7.10
7.09
2.71
2.70
2.69
2.35
2.34
2.33
1.88
1.87
1.86
1.84
1.83
1.57
O
N
O
3.70
2.00
2.25
2.03
4.68
2.12
f1 (ppm)

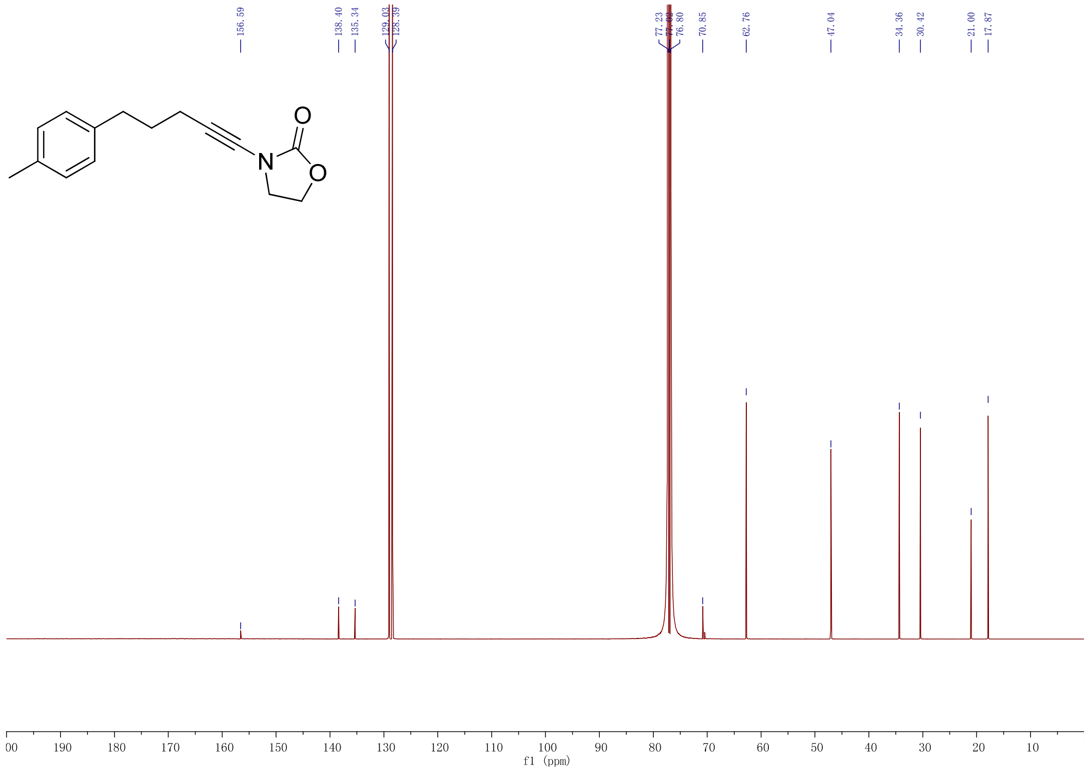
156.59
138.40
135.34
129.03
128.39
77.23
76.80
70.85
62.76
47.04
34.36
30.42
21.00
17.87
O
N
O
f1 (ppm)

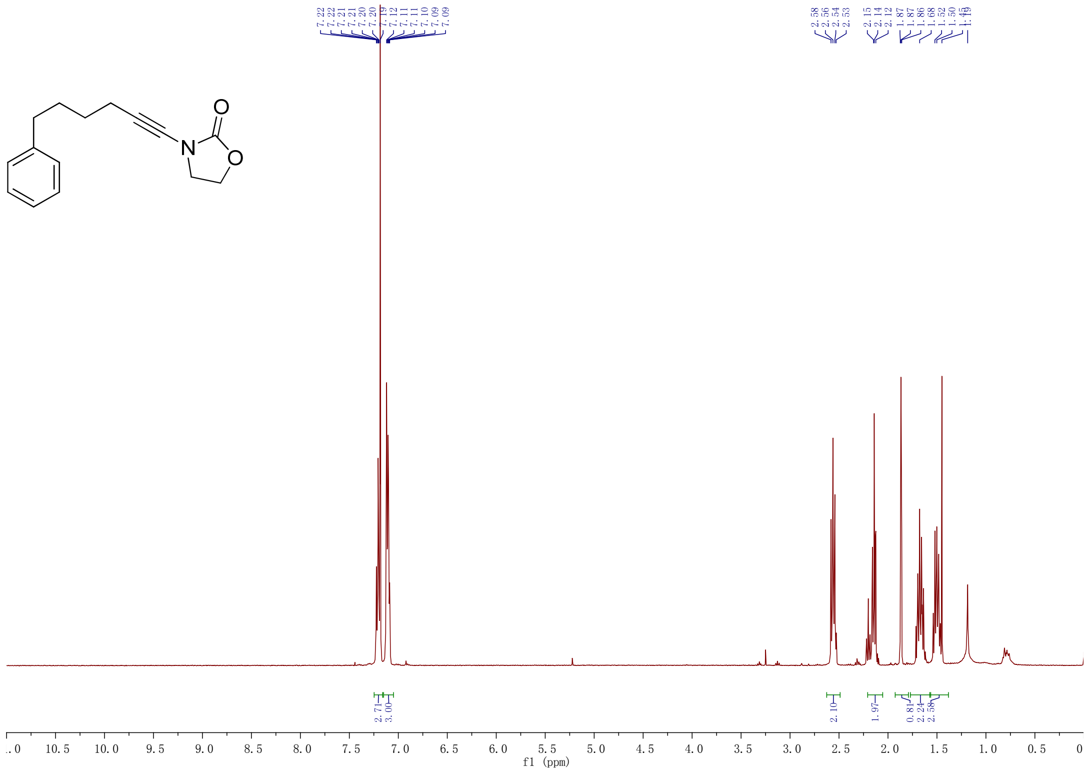

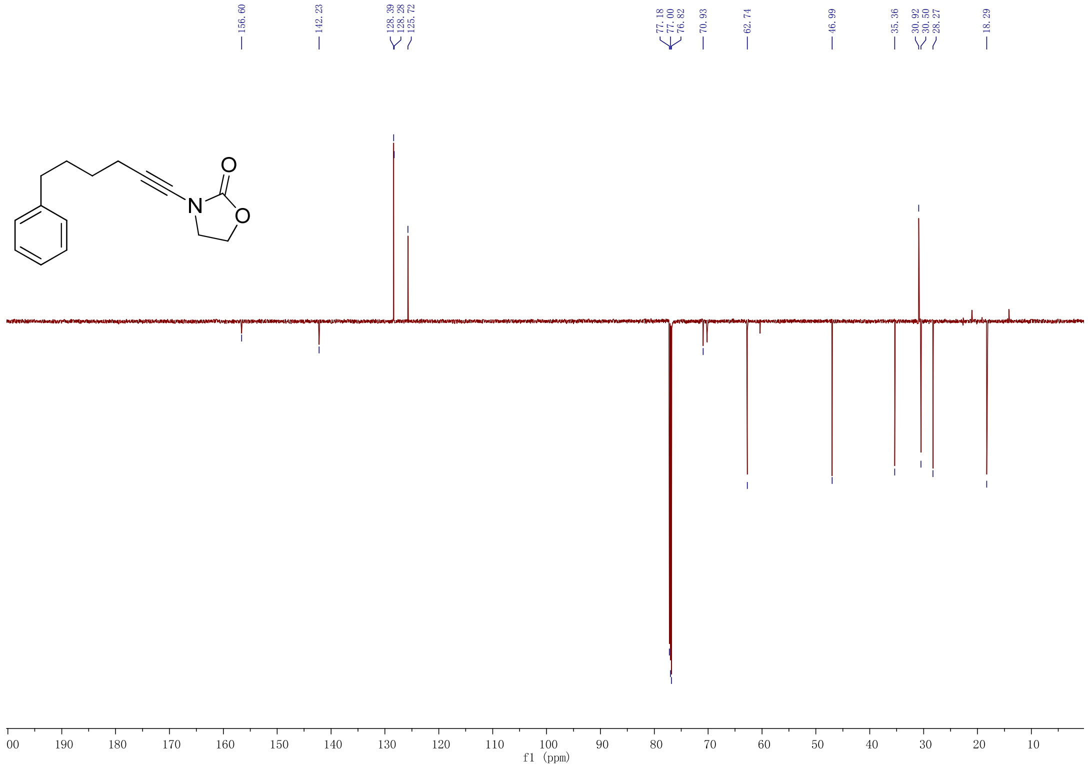

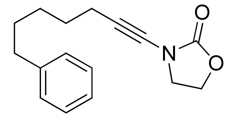
O
N
O

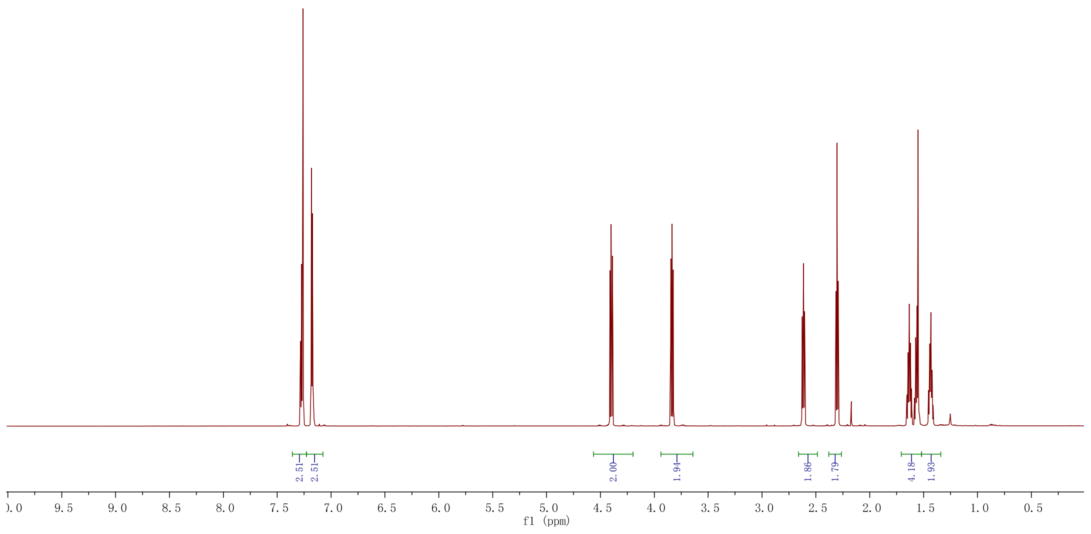
7.28
7.27
7.27
7.26
7.18
7.17
7.16
4.41
4.40
4.40
4.39
3.85
3.84
3.83
3.82
2.63
2.62
2.60
2.31
2.30
2.29
2.17
1.66
1.64
1.63
1.62
1.61
1.58
1.57
1.56
1.55
1.54
1.45
1.45
1.44
1.44
1.43
1.42
1.42
1.41
2.51
2.51
2.00
1.94
1.86
1.79
4.18
1.93
f1 (ppm)

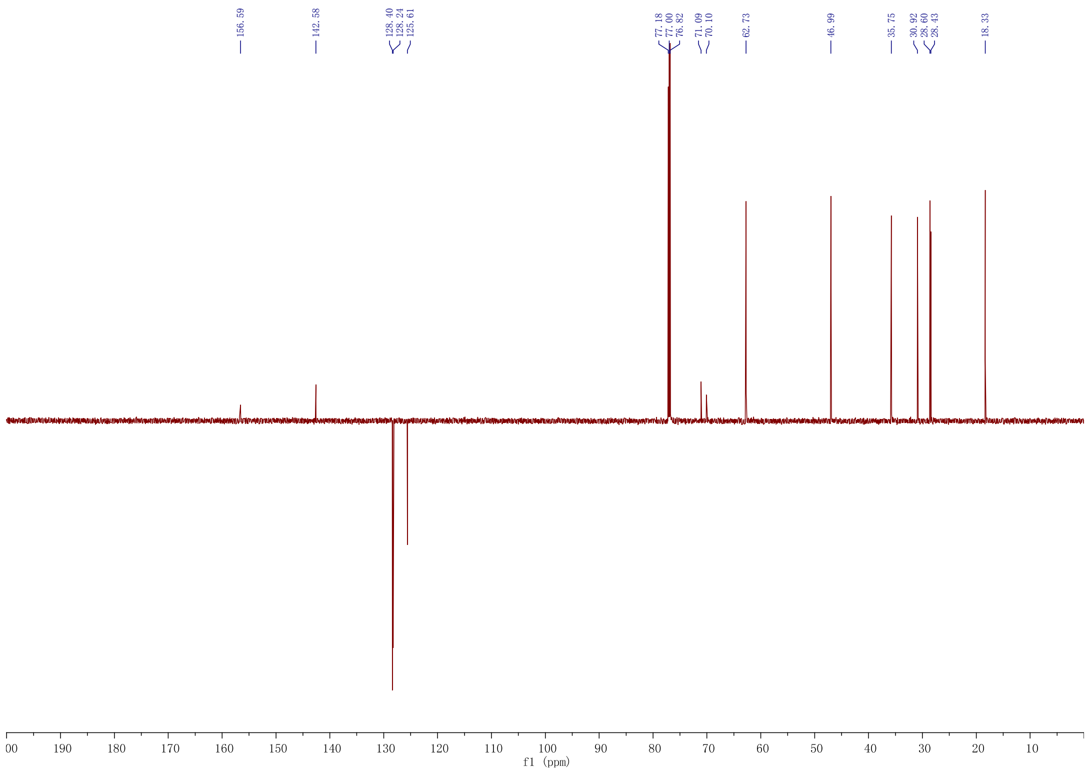
156.59
142.58
128.40
128.24
125.61
77.18
77.00
76.82
71.09
70.10
62.73
46.99
35.75
30.92
28.60
28.43
18.33
f1 (ppm)

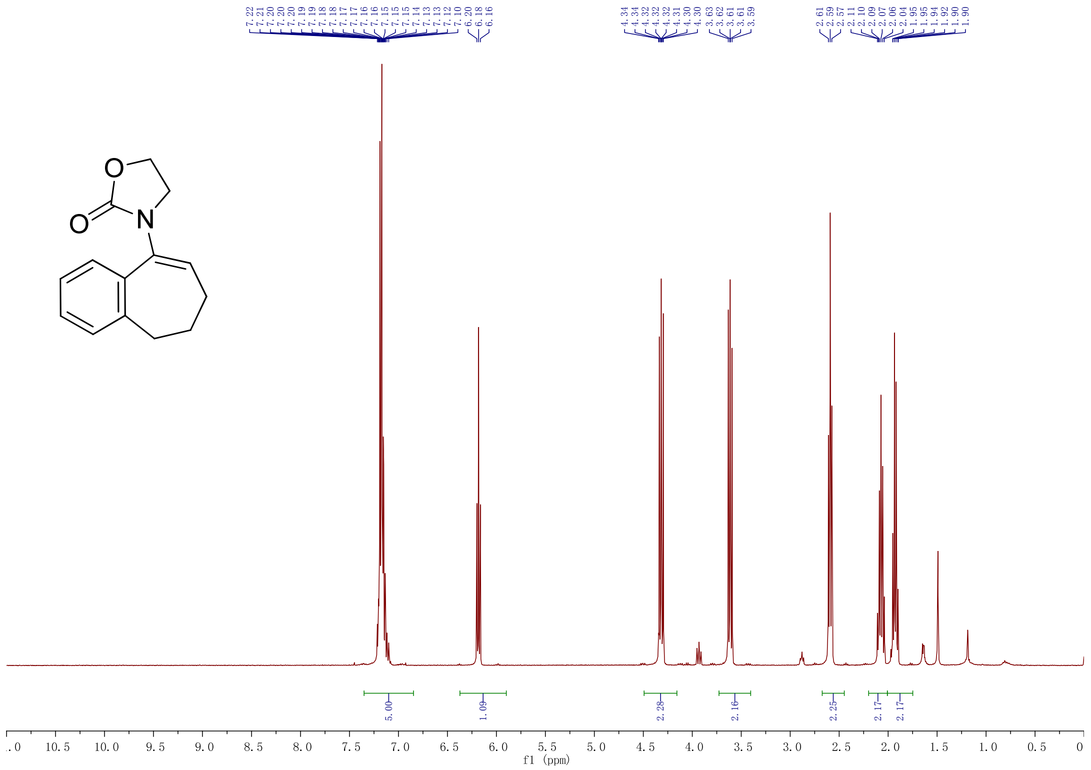

O
N
O
7.22 7.21 7.20 7.20 7.19 7.19 7.18 7.18 7.17 7.17 7.16 7.16 7.15 7.15 7.15 7.14 7.13 7.13 7.12 7.10 6.20 6.18 6.16
4.34 4.34 4.32 4.32 4.32 4.31 4.30 4.30 3.63 3.62 3.61 3.61 3.59
2.61 2.59 2.57 2.11 2.10 2.09 2.07 2.06 2.04 1.95 1.95 1.94 1.92 1.90 1.90
5.00
1.09
2.28
2.16
2.25
2.17
2.17
f1 (ppm)

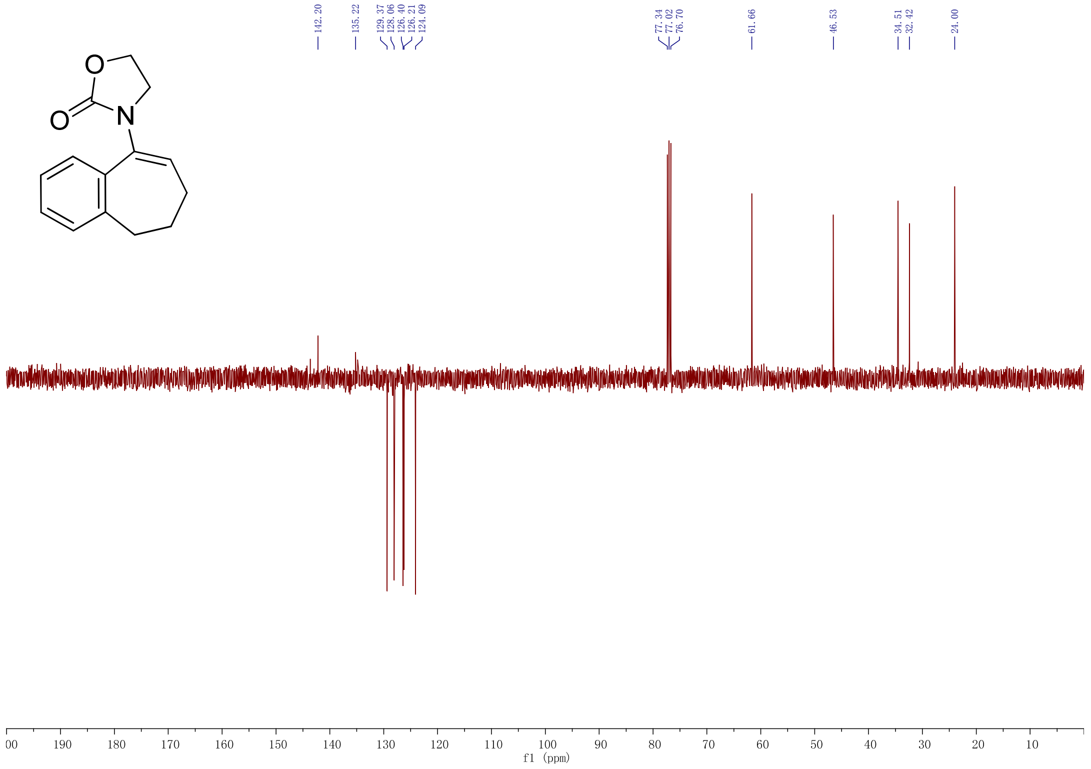

O
N
O
142.20
135.22
129.37 128.06 126.40 126.21 124.09
77.34 77.02 76.70
61.66
46.53
34.51 32.42
24.00
f1 (ppm)

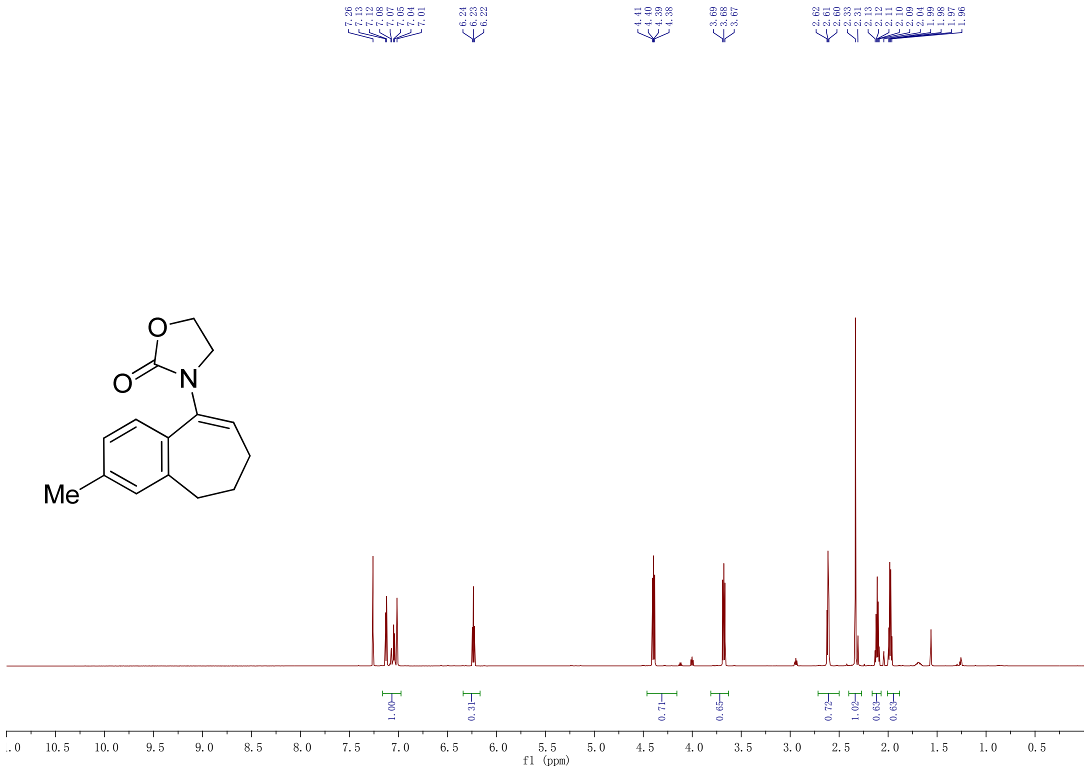
7.26
7.13
7.12
7.08
7.07
7.05
7.04
7.01
6.24
6.23
6.22
4.41
4.40
4.39
4.38
3.69
3.68
3.67
2.62
2.61
2.60
2.33
2.31
2.13
2.12
2.11
2.10
2.09
2.04
1.99
1.98
1.97
1.96
O
O
N
Me
1.00
0.31
0.71
0.65
0.72
1.02
0.63
0.63
f1 (ppm)

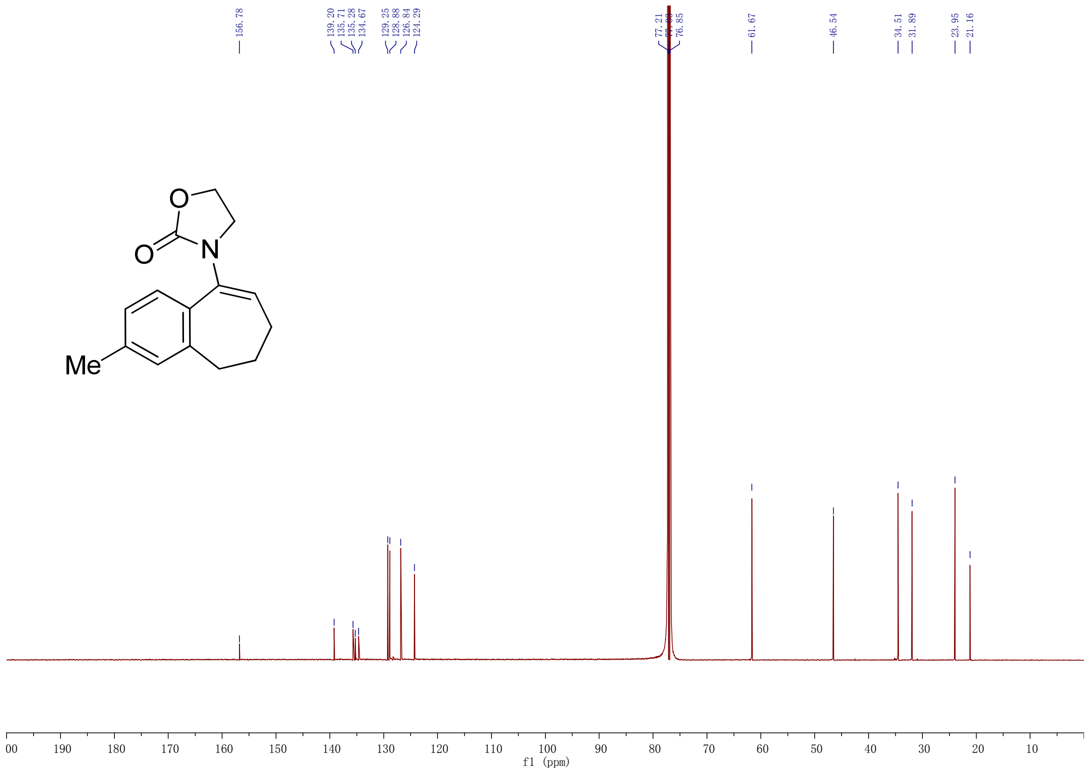
156.78
139.20
135.71
135.28
134.67
129.25
128.88
126.84
124.29
77.21
76.85
61.67
46.54
34.51
31.89
23.95
21.16
O
O
N
Me
f1 (ppm)

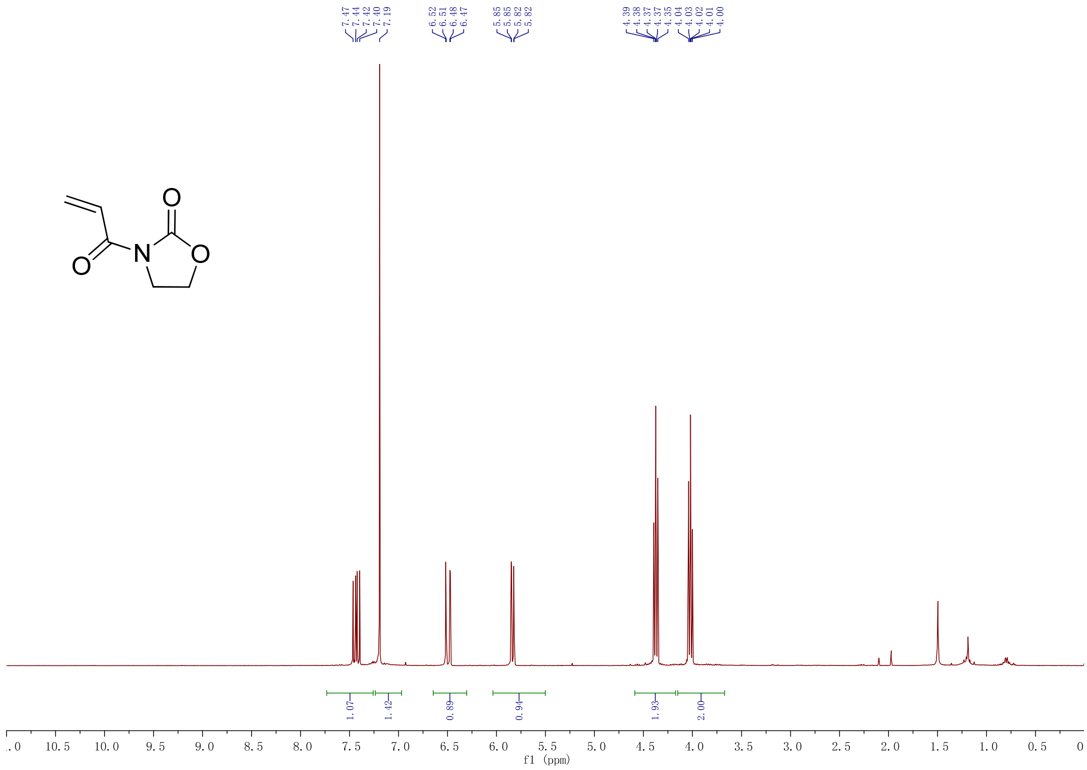
7.47
7.44
7.42
7.40
7.19
6.52
6.51
6.48
6.47
5.85
5.85
5.82
5.82
4.39
4.38
4.37
4.35
4.04
4.03
4.02
4.01
4.00
O
N
O
O
1.07
1.42
0.89
0.94
1.93
2.00
f1 (ppm)

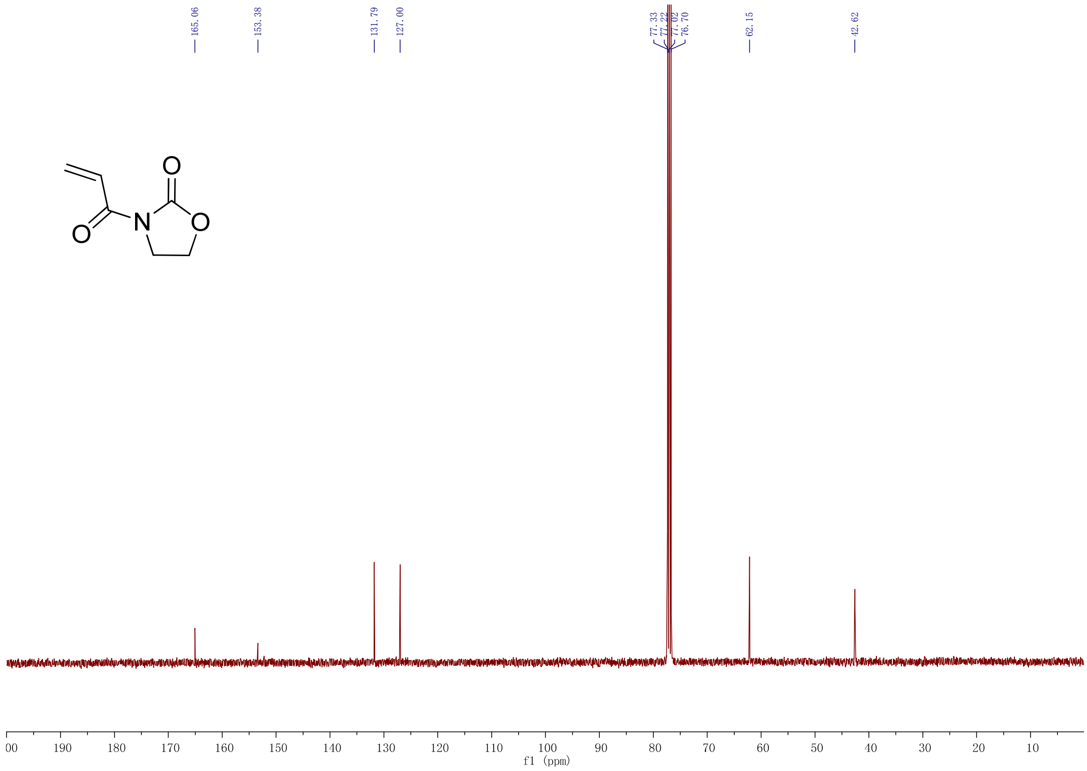
165.06
153.38
131.79
127.00
77.33
77.22
77.02
76.70
62.15
42.62
O
N
O
O
f1 (ppm)
